# Outbreaks by canopy-feeding geometrid moth cause state-dependent shifts in understorey plant communities

**DOI:** 10.1007/s00442-013-2648-1

**Published:** 2013-04-09

**Authors:** Stein Rune Karlsen, Jane Uhd Jepsen, Arvid Odland, Rolf Anker Ims, Arve Elvebakk

**Affiliations:** 1 Norut, Northern Research Institute Tromsø, Tromsø Science Park, P.O. Box 6434, 9294 Tromsø, Norway; 2 Norwegian Institute for Nature Research, 9296 Tromsø, Norway; 3 Telemark University College, 3800 Bø, Norway; 4 Department of Arctic and Marine Biology, University of Tromsø, 9294 Tromsø, Norway; 5 Tromsø University Museum, University of Tromsø, 9037 Tromsø, Norway

**Keywords:** Plant-herbivore interaction, *Empetrum nigrum*, *Avenella flexuosa*, Birch, Tundra-forest ecotone

## Abstract

The increased spread of insect outbreaks is among the most severe impacts of climate warming predicted for northern boreal forest ecosystems. Compound disturbances by insect herbivores can cause sharp transitions between vegetation states with implications for ecosystem productivity and climate feedbacks. By analysing vegetation plots prior to and immediately after a severe and widespread outbreak by geometrid moths in the birch forest-tundra ecotone, we document a shift in forest understorey community composition in response to the moth outbreak. Prior to the moth outbreak, the plots divided into two oligotrophic and one eutrophic plant community. The moth outbreak caused a vegetation state shift in the two oligotrophic communities, but only minor changes in the eutrophic community. In the spatially most widespread communities, oligotrophic dwarf shrub birch forest, dominance by the allelopathic dwarf shrub *Empetrum nigrum* ssp. *hermaphroditum*, was effectively broken and replaced by a community dominated by the graminoid *Avenella flexuosa*, in a manner qualitatively similar to the effect of wild fires in *E. nigrum* communities in coniferous boreal forest further south. As dominance by *E. nigrum* is associated with retrogressive succession the observed vegetation state shift has widespread implications for ecosystem productivity on a regional scale. Our findings reveal that the impact of moth outbreaks on the northern boreal birch forest system is highly initial-state dependent, and that the widespread oligotrophic communities have a low resistance to such disturbances. This provides a case for the notion that climate impacts on arctic and northern boreal vegetation may take place most abruptly when conveyed by changed dynamics of irruptive herbivores.

**Electronic supplementary material:**

The online version of this article (doi:10.1007/s00442-013-2648-1) contains supplementary material, which is available to authorized users.

## Introduction

Top-down trophic control of vegetation by herbivores can effectively change the state of the vegetation and cause dramatic shifts between alternative dynamic regimes (Scheffer and Carpenter 2003; Schmitz 2004; Schmitz et al. 2006). Grazing and trampling by large and medium-sized herbivores change ecosystem functioning by affecting the soil biota (Sørensen et al. 2009), nutrient cycling and availability (Stark et al. 2003; Olofsson et al. 2004) and plant abundance and diversity (Manier and Hobbs 2006; Bråthen et al. 2007). In the tundra, for instance, grazing by reindeer has been shown to reduce the cover of both ground lichens (van der Wal et al. 2001; den Herder et al. 2003; Johansen and Karlsen 2005) and dwarf shrubs (den Herder et al. 2008; Olofsson et al. 2009) and has been hypothesized to drive predictable step-wise transitions from unproductive lichen-dominated vegetation to more productive moss- and graminoid-dominated vegetation types (Oksanen and Oksanen 2000; Zimov 2005; van der Wal 2006). However, the likelihood of such transitions, and expectations on the specific nature of their trajectories, are likely to be context-dependent. First, spatially heterogeneous edaphic conditions typically shape the initial state of the vegetation in terms of dominant growth forms with different tolerance to herbivory (Proulx and Mazumder 1998; Wookey et al. 2009; Ravolainen et al. 2010; Speed et al. 2010). Thus the resistance of vegetation to herbivore impacts can be expected to be initial-state dependent. Second, herbivore impact differs in mode and strength depending on a multitude of herbivore taxon-specific traits, for instance, determining their population dynamics and food plant preferences.

In contrast to the more sustained impact of grazing by large and medium-sized herbivores, outbreaks by cyclic insect herbivores take the form of rapid, catastrophic disturbance events that can cause sharp transitions between vegetation states. Compound disturbances by insect outbreaks may change, accelerate, or delay successional pathways (McCullough et al. 1998; Shorohova et al. 2009; Man and Rice 2010), leading for instance to an effective rejuvenation of the vegetation. An example of this is epidemic outbreaks by mountain pine beetle in stands of mature lodgepole pine (*Pinus contorta*), an early successional tree species (Cattelino et al. 1979). Despite inflicting massive mortality in mature stands, the beetle is an important agent in maintaining continued stands of lodgepole pine, as the build-up of dead wood in the forest following beetle attack mediates wild fires, which are a prerequisite for opening the serotinous lodgepole cones, and hence for lodgepole regeneration (Raffa and Berryman 1987; Malmstrom and Raffa 2000). In a different system, however, attacks by the same beetle species is threatening to drive the less-adapted whitebark pine (*Pinus albicaulis*) to the brink of extinction (Logan et al. 2010), illustrating how top-down effects of insect outbreaks may also invoke regime shifts (Scheffer and Carpenter 2003; Jasinski and Payette 2005) with profound consequences for the entire trophic system (Schmitz et al. 2006; McKinney et al. 2009; Logan et al. 2010; Tomback and Achuff 2010). While such examples of pervasive ecosystem effects of insect outbreaks usually regard insects specialized on certain tree species and thus primary cascades from their direct impacts on the tree layer in forest ecosystems, some boreal outbreaking insects have a more generalized impact that even extend into tundra ecosystems (Post and Pedersen 2008).

In the birch forest-tundra ecotone in northern Fennoscandia, outbreaks by several species of spring-feeding geometrid moth occur at approximately decadal intervals (Tenow 1972), leading to local or regional canopy defoliation of the preferred host tree, mountain birch (*Betula pubescens* ssp. *tortuosa*), as well as certain understorey species. Geometrid population outbreaks dominates the natural disturbance regime in the region in the absence of large-scale wild fires, and recurring outbreaks have been documented as far back as historical records go (to the 1860s; Tenow 1972). Hence, the resilience of the mountain birch forest system to moth outbreaks is thought to be high. However, high grazing pressure by large herbivores in the damaged areas can trigger a shift in the state of the vegetation from birch forest to tundra (Kallio and Lehtonen 1973; Lehtonen 1987; Chapin et al. 2004). Moreover, the birch forest geometrids are highly climate sensitive (Bylund 1999) and for that sake their outbreaks have been predicted to become more extensive and/or severe with climatic warming (Callaghan et al. 2004). Recent studies support these predictions (Jepsen et al. 2008, 2011). The most recent (2002–2009) moth outbreak in northern Fennoscandia was of historically unprecedented extent and severity, and is likely to have been accentuated by recent outbreak range expansions of the two moth species involved (Hagen et al. 2007; Jepsen et al. 2008; Post et al. 2009). In the course of the outbreak as much as one third or 10,000 km^2^ of the birch forest in northern Fennoscandia was affected by severe defoliation in 1 or more years (Jepsen et al. 2009).

**Fig. 1:**
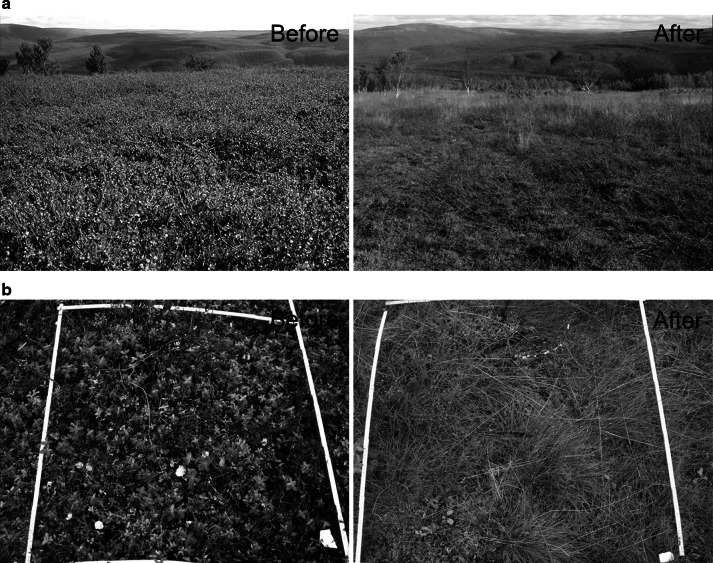
Photographs illustrating the effects that a severe outbreak of geometrid moth may have on birch forest plant communities. **a** *Betula nana*-dominated open heath site located immediately above the tree line before (16 August 2002) and after (22 August 2006) the moth outbreak. **b** *Chamaepericlymenum suecicum*-dominated forest plot before (29 August 2002) and after (19 August 2006) the moth outbreak

While the effect of moth outbreaks on die-back and regeneration of the main host, birch, has received some attention in the past (Lehtonen 1987; Hoogesteger and Karlsson 1992; Lehtonen and Heikkinen 1995; Tenow et al. 2004), vegetation changes in the herb and dwarf shrub layer of the forest floor following moth outbreaks have been poorly documented (but see Lehtonen and Yli-Rekola 1979; Jepsen et al. 2013). Generally, the understorey of northern boreal forest tends to have received less focus than the tree layer despite the fact that their primary productivities are comparable and that ecosystem functions provided by understorey plants is crucial for the integrity of these ecosystems (Nilsson and Wardle 2005). During an outbreak cycle, moth larval density in the canopy may change from barely detectable levels to several hundred larvae per branch (Hogstad 2005; Klemola et al. 2008; Jepsen et al. 2009), causing complete depletion of the birch canopy early in the season (Tenow 1972). Under such circumstances larvae have also been reported to feed on several deciduous understorey species, including dwarf birch *Betula nana* (Fig. 1) and bilberry *Vaccinium myrtillus* (e.g. Kallio and Lehtonen 1973; Lehtonen and Yli-Rekola 1979). Mass occurrences of larvae can, however, also be expected to dramatically alter the conditions for a number of understorey plant species not affected by direct defoliation, most noticeably by a change in light conditions (as the canopy is reduced), added nutrients (from larvae droppings and decomposing larvae carcasses), and altered recycling of nutrients and reduced root competition (both from root die-back). This in turn can be expected to change the understorey vegetation state through processes that involve the combination of plant resistance to change in biotic interactions (herbivory and interactions between plant species) and abiotic influences (nutrients and light). As these northern birch forests constitute mosaics of different understorey plant communities based on contrasting edaphic conditions (Hämet-Ahti 1963) it could also be expected that the initial vegetation state could mould the impact of insect outbreaks.

In the current study we investigate the changes in understorey vegetation following the most recent outbreak by birch forest geometrid moth in northern Fennoscandia. We focus our study on the Varanger Peninsula in northern Norway which is located in the birch forest-tundra ecotone and encompass the north-eastern periphery of the outbreak zone. Incidentally, a quantitative survey of the understorey vegetation had been undertaken the year before the outbreak commenced. This initial survey encompassed sites both within and outside the geometrid outbreak area, which we could exploit by re-surveying these sites towards the end of the outbreak period 4–5 years later to provide a powerful assessment of the impact of the outbreak. The specific objectives of our study were to: (1) determine if moth outbreaks caused a shift in birch forest understorey community composition, (2) determine the extent to which such shifts in community composition were dependent on the state of the community prior to the outbreak, and (3) determine whether indirect effects of the moth outbreak could be detected through changes in abundances of non-host plant species.

## Materials and methods

### Study region

The study was conducted in four areas situated on the Varanger Peninsula, which is the north-easternmost part of mainland Norway, between 70–71°N and 28–31°E. The peninsula is located in the arctic-boreal transition zone (Elvebakk et al. 1999; Karlsen et al. 2005) with a mean July temperature ranging from about 10 °C at the north-eastern coast to above 12 °C in the south-western interior, and where the annual precipitation is in the range 356–660 mm (www.met.no). The length of the growing season, defined as the period from onset of leafing of birch to 50 % yellowing of leaves of birch, is between 100 and 120 days (Karlsen et al. 2008, 2009). The bedrock geology of the peninsula consists of sedimentary rocks of the Late Proterozoic to Early Cambrian age (Siedlecka and Roberts 1992; Sochava and Siedlecka 1997), which creates a variety of nutrient conditions. Birch (*Betula pubescens*) is the dominant tree species, and covers large areas in the south-western parts of the peninsula, as well as in protected valleys in northern and eastern parts. On dry and nutrient-poor ground a birch forest type occurs which is characterized by dominance of *Empetrum nigrum* ssp. *hermaphroditum,* *Vaccinium myrtillus* and *Chamaepericlymenum suecicum* in the field layer, and on slightly more mesic ground *E. nigrum* becomes less pronounced (Hämet-Ahti 1963; Virtanen et al. 1999; Karlsen et al. 2005). A variety of a more nutrient-demanding herb-rich birch forest type occurs in a more scattered manner, while on the driest and most oligotrophic sites *E. nigrum*-lichen (*Cladina* spp.)-dominated forest types occur. Riparian forests, characterized by *Salix* spp. dominance in the tree and shrub layer, and a highly variable field layer, can be common locally along rivers.

### Moth outbreak history in the study region

Recurring population outbreaks by the two geometrids, autumnal moth (*Epirrita autumnata* Bkh.) and winter moth (*Operophtera brumata* L.), dominate the natural disturbance regime in the Fennoscandian birch forest. Both moth species exhibit cyclic population outbreaks at approximately 10-year intervals in this region (Tenow 1972; Bylund 1999; Neuvonen et al. 1999). The cyclicity of the outbreaks is documented in qualitative historical records as far back as the 1860s (Tenow 1972; Nilssen et al. 2007). The outbreaks can be massive and cause severe defoliation over vast areas and occasionally death of the forest (Tenow 1972; Lehtonen and Heikkinen 1995; Tenow and Bylund 2000). Birch is the main host tree of both moth species in the region, but both winter moth and autumnal moth are reported to occur on a variety of host plants within their distribution ranges (Tenow 1972 and references therein). In the birch forest of Northern Fennoscandia other canopy-forming species such as the sparsely occurring *Sorbus aucuparia* and *Salix* sp. may also be defoliated when larval densities are high (Tenow 1972; Kallio and Lehtonen 1973). Similarly several understorey dwarf shrub species, in particular *B. nana* (Fig. 1), *V. myrtillus* and occasionally *Vaccinium uligonosum* may be defoliated by the larvae (Kallio and Lehtonen 1973). In parts of its distribution range (e.g. the UK) the winter moth utilizes both coniferous species such as sitka spruce (*Picea sitchensis*) and common heather (*Calluna vulgaris*) (Vanbergen et al. 2003). *E. nigrum*, however, has to the best of our knowledge never been reported as a potential host plant for either moth species.

The most current (2002–2009) outbreak cycle has been of historically unprecedented severity in northernmost Fennoscandia, and the Varanger Peninsula is the region most severely affected. A climate-mediated northeastern expansion in the outbreak range of winter moth (Jepsen et al. 2008) is thought to be part of the explanation. While the Varanger region historically has suffered outbreaks by autumnal moth, the most current outbreak involved both species with a 1- to 2-year lag between population peaks. As a result, many areas in the southern part of the Varanger Peninsula were first defoliated by autumnal moth (2002–2004) and subsequently by winter moth (2005–2006). This pattern has been documented in both field records of larval densities (Klemola et al. 2008) and by mapping of crown defoliation using remote sensing (Jepsen et al. 2009).

### Field vegetation surveys

Field vegetation surveys were done before (2001–2002) (Karlsen et al. 2004, 2005) and after (2006–2007) the geometrid moth outbreak following a hierarchical design. Four study areas with contrasting outbreak history were used: Austertana (70°27″N, 28°35″E) had a continuous outbreak from 2003 to 2006, with a peak in the extent of defoliation in 2004, while Klubbvik (70°8″N, 29°7″E) experienced two peaks in defoliation, in 2003 (autumnal moth) and 2005–2006 (winter moth). The remaining two areas, which are located in forest patches beyond (Båtsfjord, 70°35″N, 29°39″E) or at the periphery (Vadsø, 70°4″N, 29°50″E) of the continuous forest zone, were virtually unaffected by the outbreak and serve as references. A total of 18 sites were subjectively selected in homogeneous birch forest within the four study areas (two to six sites per area). Within each site, from three to 13 sample plots, each 1 × 1 m, were randomly allocated in an approximately 5 × 5-m area. The number of sample plots per site depended on the species diversity of the vegetation with fewer plots allocated to more homogeneous sites. The cover abundance of living parts of each plant species in each sample plot was determined by visual estimates by the same observer (S. R. K.) both before and after the geometrid outbreak. All surveys were done in mid to late August to ensure comparable species cover. Each site was photographed and its position determined by global positioning system, and some plots were further permanently marked with galvanized iron bolts. This allowed an accurate relocation after the outbreak. The nomenclature follows Lid and Lid (2005) for vascular plants.

### Data analyses

**Fig. 2:**
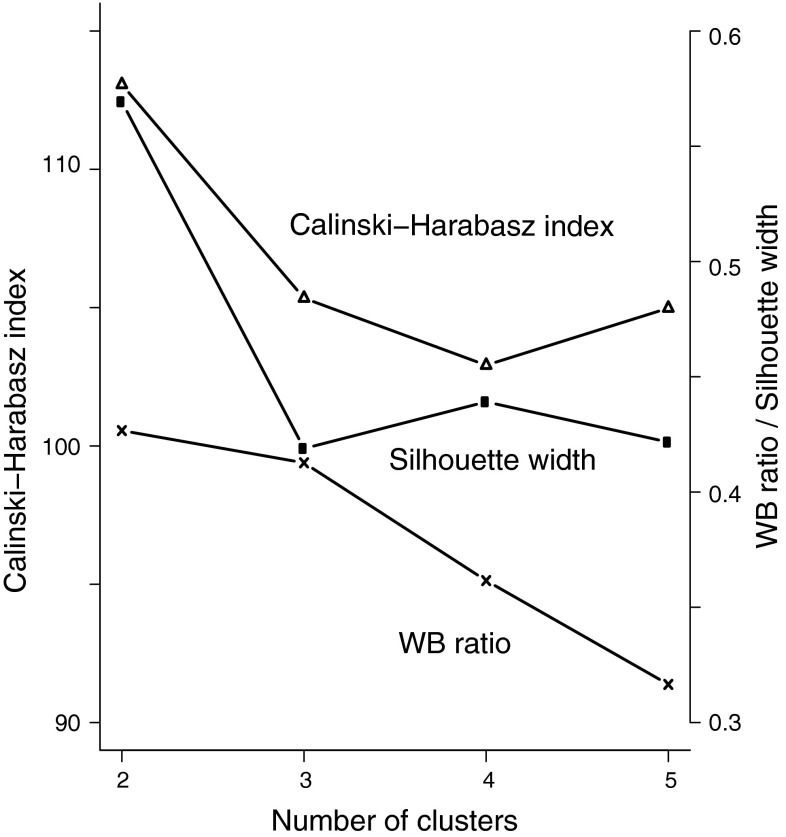
The values of a selection of cluster-validation measures inspected in order to determine the most suitable number of clusters in the dataset. The Calinski-Harabasz index and the mean silhouette width of clusters suggested that two clusters would be a suitable choice, while the ratio between the average distance within and between clusters (*WB ratio*) would suggest that there was little difference between a solution based on two and three clusters

The cover abundances of all species were organized in a plot by species matrix where each row represented a plot in a given period (Before or After the outbreak). Each plot further had associated information indicating the treatment (Reference or Outbreak depending on whether the plot was located outside or inside the outbreak range). Plant community associations prior to the outbreak (e.g. the initial states) were identified using hierarchical clustering. We used Ward’s linkage function and Morisita-Horn dissimilarities of the non-transformed cover abundance obtained before the outbreak [function hclust() in the R library stats]. The most likely number of clusters was determined by calculating a number of commonly used cluster-validation measures using cluster.stats() in the R library fpc. Most validation estimates, including the mean silhouette width and the Calinski-Harabasz index, suggested that two clusters was a suitable choice (Fig. 2), while others, including the ratio between the average distance within and between clusters (Fig. 2; WB ratio) suggested that the difference between a two-cluster and a three-cluster solution was very small. Consequently, we address both a two-cluster and a three-cluster solution in our analysis.

To identify the significant indicator species for each of the plant communities resulting from the cluster analysis, and to be able to interpret these groups in an ecological context, we performed indicator species analysis (Dufrêne and Legendre 1997) using indval() in R library vegan. Differences in community composition between the plant communities were analysed using the multi-response permutation procedure (MRPP), which is a non-parametric method for testing differences in assembly structure between a priori defined groups. Based on a dissimilarity matrix (here Morisita-Horn), MRPP calculates the observed mean within-group distance weighted by group sample size (observed δ) and the expected mean within-group distance (expected δ) based on Monte-Carlo permutation. The effect size (*A*), also referred to as the chance-corrected within-group agreement, is expressed as *A* = 1-(observed δ/expected *δ*). To visualize the change in each of the plant communities between periods (e.g. in response to the moth outbreak) we used non-metric multidimensional scaling (NMDS) ordination based on Morisita-Horn dissimilarities. NMDS was performed with multiple random starts to find the best global solution using the function metaMDS() in R library vegan. We used a three-dimensional solution since this resulted in a lower stress value (stress 0.09) than a two-dimensional solution (stress 0.14). The point configurations in the two-dimensional and three-dimensional solutions were, however, very similar. MRPP was used to test the significance of the observed shifts in plant communities between periods. To further investigate the significance of the shift in plant communities between periods and treatments we tested the impact of period (before or after the outbreak), treatment (reference or outbreak plot) and the period × treatment interaction using NMDS plot scores on each of the three ordination axis as response variables. For this we used linear mixed-effect models (LMEs) which allows for including random effects to control for autocorrelation in the data caused by nested designs. In our case the 92 plots were nested within 18 different sites, and we consequently included site as a random effect in both models. We specifically looked for significant period × treatment interactions which would indicate that outbreak plots were affected differently than reference plots by the moth outbreak. LMEs were developed using glmmadmb() in R library glmmADMB.

## Results

### Classification and characterization of initial plant community states

Hierarchical clustering of the vegetation community data using two clusters resulted in clusters containing 64 and 28 plots, respectively. The strongest indicator species for the first division of the dendrogram (e.g. into two clusters; Table 1) were *V. myrtillus, E. nigrum* and *C. suecicum* (cluster 1) and *Ranunculus acris, Geranium sylvaticum* and *Solidago virgaurea* (cluster 2), suggesting that this division separates a poorer dwarf shrub community (cluster 1) from a richer herb-rich community (cluster 2). Clustering using three clusters subdivided cluster 1 into two clusters (clusters 1.1 and 1.2; Table 1) containing 30 and 34 plots, respectively. The list of significant indicator species suggests that clusters 1.1 and 1.2 both represent dwarf shrub-dominated birch forest types, but cluster 1.1 is indicated most strongly by *E. nigrum*, suggesting a more nutrient-poor and/or drier community than cluster 1.2, which is indicated by *V. myrtillus* and *C. suecicum* (see Table 1 for a full list). *V. myrtillus* and *C. suecicum* also have relatively high indicator values for cluster 1.1, which is hence best described as a “crowberry-bilberry-dwarf cornel” type (henceforth “crowberry birch forest”). Cluster 1.2 is best described as a “bilberry-dwarf cornel” type (henceforth “bilberry birch forest”). Cluster 2 represents a herb- and grass-dominated birch forest type with a richer plant community and large number of significant indicator species identified (Table 1). In the following we term cluster 2 “low-herb birch forest”. A complementary description of the three plant communities is available in Online Resource 1 in the Electronic Supplementary Material. The three communities differed in community composition (MRPP for difference between clusters, *A* = 0.482, *P* < 0.001).

**Table 1:** Indicator values for all species that are significant indicators of the plant communities identified by hierarchical clustering

Species	Two clusters		Three clusters		
	Cluster 1	Cluster 2	Cluster 1.1	Cluster 1.2	Cluster 2
*Alchemilla glomerulans*	0	0.357^a^	0	0	0.357^a^
*Anthoxanthum nipponicum*	0	0.781^a^	0	0	0.776^a^
*Astragalus alpinus*	0	0.321^a^	0	0	0.321^a^
Bryophytes	0.558^a^	0.349	0.556^a^	0.174	0.209
*Bistorta vivipara*	0	0.786^a^	0	0	0.786^a^
*Cerastium fontanum*	0	0.464^a^	0	0	0.464^a^
*Cirsium heterophyllum*	0	0.143^a^	0	0	0.143^a^
*Calamagrostis phragmitoides*	0	0.286^a^	0	0	0.286
*Chamaepericlymenum suecicum*	0.878^a^	0.041	0.296	0.625^a^	0.022
*Empetrum nigrum*	0.824^a^	0.007	0.800^a^	0.133	0.003
*Equisetum pratense*	n.s.	n.s.	0	0.147^a^	0
*Festuca rubra*	0	0.566^a^	0	0.001	0.562^a^
*Filipendula ulmaria*	0	0.179^a^	0	0	0.179^a^
*Geum rivale*	0	0.214^a^	0	0	0.214^a^
*Geranium sylvaticum*	0	0.999	0	0	0.998^a^
*Hieracium* sp.	0.001	0.520^a^	0	0.003	0.507^a^
*Lycopodium annotinum*	0	0.143^a^	0	0	0.143^a^
*Linnea borealis*	0.188^a^	0	0.174^a^	0.041	0
*Luzula pilosa*	0.016	0.400^a^	0	0.046	0.327^a^
*Melampyrum pratense*	0.281^a^	0	0.071	0.227^a^	0
*Orthilia secunda*	0	0.321^a^	0	0	0.321^a^
*Pyrola minor*	0	0.607^a^	0	0	0.607^a^
*Parnassia palustris*	0	0.214^a^	0	0	0.214^a^
*Rumex acetosa*	0.001	0.169^a^	0	0.003	0.162^a^
*Ranunculus acris*	0	1.000^a^	0	0	1.000^a^
*Rubus saxatilis*	0	0.643^a^	0	0	0.643^a^
*Saussurea alpina*	0	0.143^a^	0	0	0.143^a^
*Salix glauca*	0	0.107^a^	0	0	0.107^a^
*Selaginella selaginoides*	0	0.286^a^	0	0	0.286^a^
*Solidago virgaurea*	0.002	0.963^a^	0.003	0.002	0.928^a^
*Trientalis europaea*	0.001	0.170^a^	0	0.002	0.164^a^
*Trollius europaeus*	0	0.286^a^	0	0	0.286^a^
*Viola biflora*	0	0.964^a^	0	0	0.964^a^
*Vaccinium myrtillus*	0.886^a^	0.046	0.323	0.608^a^	0.024

Results are shown for a two-cluster solution and a three-cluster solution

^a^Highest indicator value per species

### Moth outbreak-induced shifts in community composition and species abundances

**Fig. 3:**
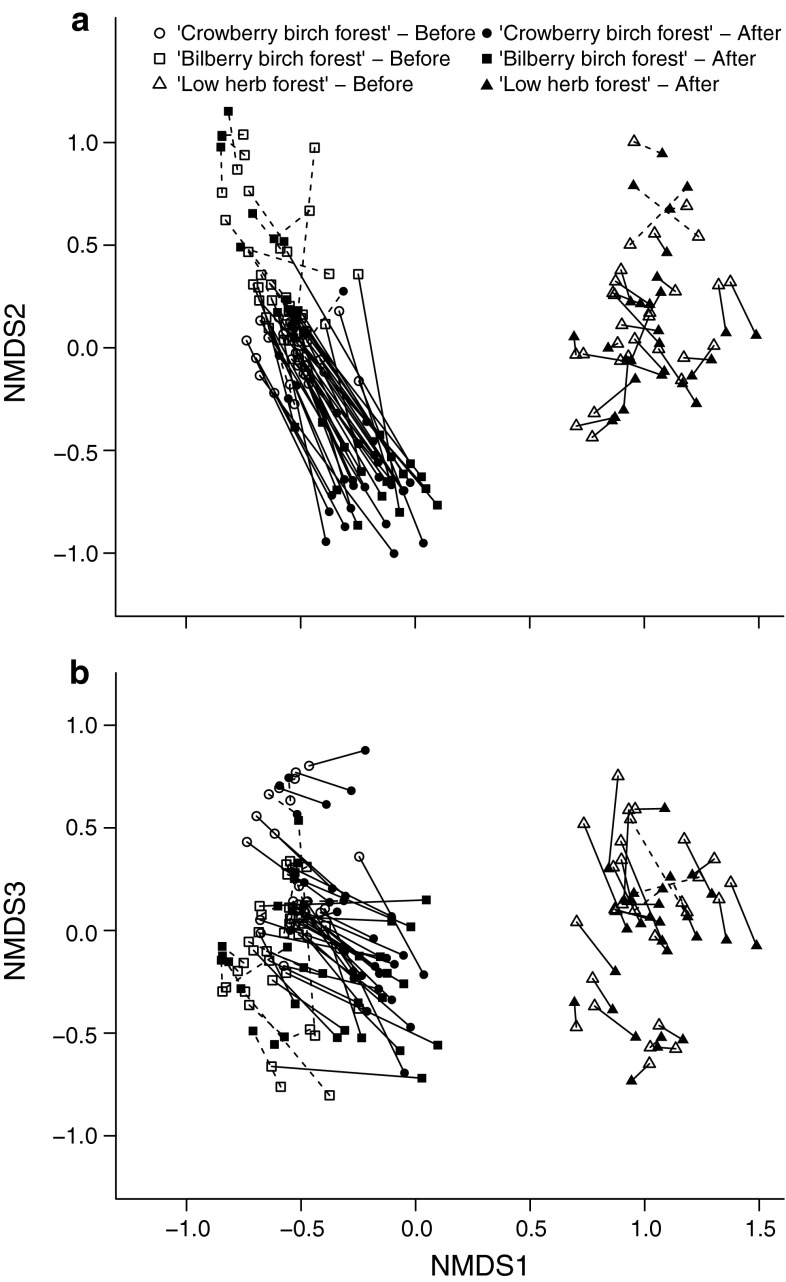
Nonmetric multidimensional scaling (*NMDS*) ordination of the 92 plots before (*open symbols*) and after (*closed symbols*) the moth outbreak. While the two dwarf shrub communities show marked shifts along the 2nd axis, the low-herb community shows only non-directional shifts in the same magnitude as the reference plots. *Black lines* connect the before-after ordination scores of plots located within the outbreak range, while* dotted lines* connect the before-after score of reference plots located outside the outbreak range. Stress value of the three-dimensional solution: 0.14

**Fig. 4:**
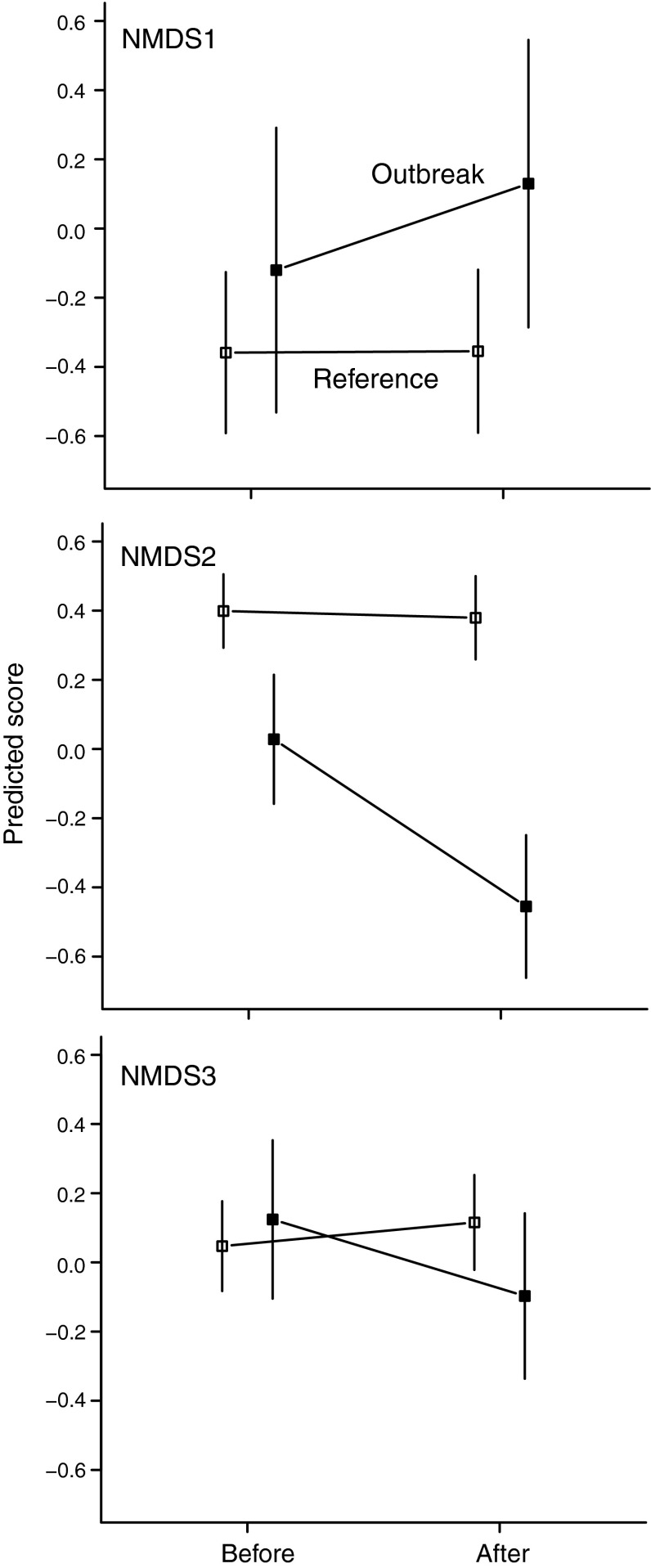
Linear mixed-effect model predictions with SEs for the moth outbreak-induced shift in community composition along the three NMDS ordination axes (NMDS1-3) for outbreak (*filled symbols*) and reference (*open symbols*) plots

The understorey community composition shifted markedly in response to the moth outbreak (MRPP for difference between periods, all plots, *A* = 0.066, *P* < 0.001; outbreak plots only, *A* = 0.16, *P* < 0.001). Based on the NMDS ordination (Fig. 3), it is clear that not all communities contributed to this shift, showing that plant community resistance to moth outbreaks differed widely depending on the initial state of the vegetation. Plots located in the two more oligotrophic dwarf shrub communities showed large and directed shifts primarily along the 2nd axis, while plots in the more eutrophic low-herb community, showed much smaller, non-directional shifts of a similar magnitude to the reference plots (Fig. 3). Linear mixed-effects models developed using the NMDS ordination scores as response variables and site as a random effect (Fig. 4) confirm that community changes in response to the outbreak differs between outbreak and reference plots, as indicated by highly significant period × treatment interactions for all three ordination axis (NMDS1, *z* = 5.54, *P* < 0.001; NMDS2, *z* = 6.92, *P* < 0.001; NMDS3, *z* = 5.60, *P* < 0.001).

**Fig. 5:**
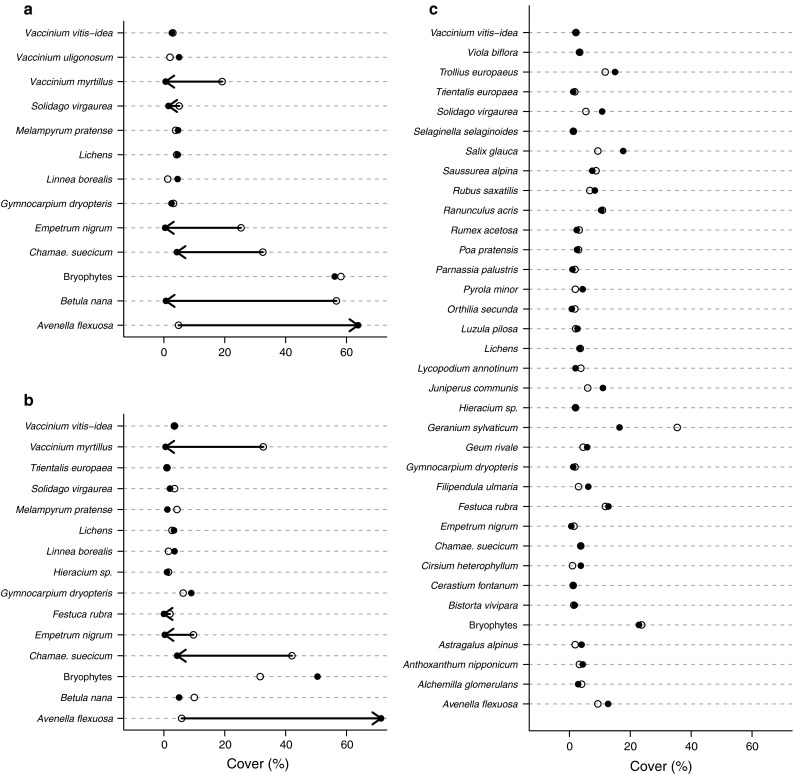
The cover abundance of individual species before (*open symbols*) and after (*closed symbols*) the moth outbreak. Cover abundances are given as mean for all plots in which a given species is present within a given community. **a** Crowberry birch forest, **b** bilberry birch forest, **c** low-herb birch forest. Cover abundances with non-overlapping SDs before and after the outbreak are connected by* arrows*. *Chamae. suecicum* *Chamaepericlymenum suecicum*

The significant shifts in understorey community composition observed in the two oligotrophic communities were mainly due to large changes in abundance of a limited number of plant species (Fig. 5). The two oligotrophic communities experienced a drastic transition from dominance by dwarf shrubs (*V. myrtillus*, *B. nana* and *E. nigrum*) to dominance by the grass *A. flexuosa*. In addition, the low herb *C. suecicum* experienced a large decrease in cover in both communities (Fig. 5a, b). *C. suecicum* is defoliated by larvae at outbreak densities (authors’ observations). None of the species in the eutrophic community (Fig. 5c) showed significant changes in abundance in response to the moth outbreak.

## Discussion

Owing to the incidental, but fortunate, spatially extensive vegetation survey (Karlsen et al. 2004, 2005) conducted just prior to the onset of one of the most severe and large-scale insect outbreak recorded across the circumpolar forest-tundra ecotone (Post et al. 2009), we could apply a quasi-experimental approach to assess some crucial aspects of the impact of the outbreak. In particular, these circumstances allowed us to demonstrate the magnitude of the impact on community composition and individual plant species abundances, and how the impact was dependent on the initial state of the vegetation. Previous studies have reported changes in understorey plant species abundance during the years following geometrid moth outbreaks without knowledge of pre-outbreak abundances (Kallio and Lehtonen 1973; Lehtonen and Yli-Rekola 1979). The setting of the present study, however, allowed for a direct quantitative assessment and inferences about which factors make the vegetation more or less resistant to massive disturbances of this kind.

In general we found that the compound disturbances inflicted by the geometrids that defoliated the tree layer also caused major changes in the understorey vegetation. The nature and magnitude of these changes were, however, conditional on the initial state of the vegetation according to dominant growth forms (dwarf shrubs or herbs) and/or nutrient status (oligotrophic or eutrophic). The impact of the moth outbreak in the two oligotrophic dwarf shrub communities initially dominated by dwarf shrubs caused significant shifts along the main floristic gradients that ought to be characterized as a vegetation state shift. In the eutrophic community, by comparison, the change was negligible. Our results corroborate the findings of a recent study (Jepsen et al. 2013) which addressed the cascading effects of moth defoliation on plant and herbivore communities on a regional scale in northern Norway. Without knowledge of plant or herbivore abundances prior to the outbreak, the authors relied on regional contrast in defoliation severity to demonstrate a relationship between increased defoliation and increased abundance of grasses (in particular *A. flexuosa*) and decreased abundances of dwarf shrubs (in particular *E. nigrum*) in addition to significant cascading effects on the herbivore community. The results of the current study demonstrate with all clarity that the patterns inferred by Jepsen et al. (2013) from regional spatial contrasts are founded on actual temporal transitions from dwarf shrub dominance to grassland on a local scale.

The two dwarf shrub communities suffered an almost complete loss of live dwarf shrubs after the moth outbreak. Thus the low resistance of the woody growth forms in the field layer mirrors that of the birch in the tree layer, as the majority of the birch trees do not survive such intensive and long-lasting defoliation (Tenow 1972). Although birch is the main host plant for the two geometrid moth species in question, moth larvae also defoliate deciduous dwarf shrub species such as *B. nana* and *V. myrtillus* during mass outbreaks (Kallio and Lehtonen 1973; Lehtonen and Yli-Rekola 1979; Fig. 1). However, *E. nigrum* which is generally unpalatable to herbivores (Bråthen et al. 2007), appears not be defoliated (Kallio and Lehtonen 1973; Online Resource 2). Hence, the extensive mortality of this evergreen remains something of an enigma that deserves further, probably experimental, investigation. Although deciduous species are the favoured hosts, larvae of both species have been reported to defoliate evergreen trees (Tenow 1972), and winter moth can reach outbreak densities on sitka spruce and common heather (Vanbergen et al. 2003). Although no obvious foliage loss is evident in *E. nigrum* during mass outbreaks, a possibility is that starving larvae attempt to eat the leaves (e.g. punctuate them without causing visible foliage loss), thereby weakening the plant by making it more susceptible to desiccation or infection by fungal pathogens (Olofsson et al. 2011).

In both dwarf shrub communities *A. flexuosa* responded positively to the disturbance and came to completely dominate the vegetation (Figs. 1, 5). *A. flexuosa* is not defoliated by moth larvae and probably became released from limitations of light under canopies of birch shrubs and trees (that became defoliated) and nutrients that became added as larval frass and carcasses to the oligotrophic soil. It is also likely that the positive response in *A. flexuosa* is tightly linked to the loss of shrubs; in particular the allelopathic *E. nigrum* which often is the dominant of late-successional stages of heath and nutrient-poor forest vegetation (Haapaasari 1988; Tybirk et al. 2000; Bråthen et al. 2010). Dominance by *E. nigrum* is associated with retrogressive succession due to an accumulation of polyphenolic compounds in the soil (Nilsson and Wardle 2005). This in turn adversely affect soil microbial activity, decomposition rates, nutrient availability and hence tree and understorey productivity (Wardle et al. 1997; DeLuca et al. 2002; Wardle et al. 2003; Nilsson and Wardle 2005). In northern boreal coniferous and mixed forests the dominance of *E. nigrum* is broken by recurrent fires (Zackrisson et al. 1995, 1996), which both remove above-ground plant parts and change soil conditions such as pH, humus layer thickness and nutrient availability. Field experimental studies have confirmed that severe disturbance is needed to break the dominance of *E. nigrum*, and that the effects of disturbance are enhanced by fertilization (Olofsson et al. 2005; Manninen et al. 2011). We have documented here that severe moth outbreaks effectively break the dominance of *E. nigrum* in a manner qualitatively similar to wild fires in coniferous forest, albeit on a much larger spatial scale. Dwarf shrub birch forest is, by far, the most common forest type in the Tana-Varanger region where our study areas are located (Hämet-Ahti 1963; Väre 2001; Wielgolaski 2001). It here covers approximately 900 km^2^ and virtually all of this area was affected by severe defoliation in one or more years during the period 2002–2009 (Jepsen et al. 2009). For comparison, the total forest area affected by wildfires in Norway as a whole during the same period was just above 100 km^2^ (Directorate for Civil Protection and Emergency Planning, www.dsb.no).

As outlined above, the strength of the response of the initially omnipresent *A. flexuosa* in the two oligotrophic dwarf shrub communities was likely conditional on a combination of initially low nutrient levels, its own initial abundance and the abundance of the allelopathic *E. nigrum*. In the initially eutrophic plant community, the response of graminoids (*A. flexuosa* and *F. rubra*) was either weak or absent. This was most likely because grasses were initially less nutrient limited and/or because of intense competition for space and light with a high diversity of other understorey plants (e.g. forbs) not responding to the moth outbreak. Indeed, the high species diversity of the low-herb forest types may have contributed to the resistance of these communities to disturbance (Hooper et al. 2005).

### Conclusion

The spread of massive outbreaks of folivorous insects has for some time been predicted to become one of the most severe impacts of climate warming on northern boreal forest ecosystems (Neuvonen et al. 1999; Callaghan et al. 2004). In line with these predictions we have now empirically demonstrated the impact of an unprecedented geometrid moth outbreak due to a range expansion of the winter moth that extended across the birch forest-tundra ecotone in northernmost Fennoscandia (Jepsen et al. 2008; Post et al. 2009). We found that the most spatially extensive plant communities in the impacted region, two types of oligotrophic dwarf shrub birch forest, experienced large shifts in community composition and hence had very low resistance to the outbreak. This was apparently due to the initial dominance of woody growth forms (*E. nigrum* and *V. myrtillus*) that were intolerant to defoliation, and the occurrence of a widespread graminoid that had an initially low abundance, which rapidly gained dominance when nutrients became available from larval excreta and the allelopatric dwarf shrub *E*. *nigrum* was eliminated. In effect the vegetation of vast areas was transformed from a shrub-dominated state to grassland. Our finding corroborates the notion that high-latitude ecosystems may generally have little resistance to climate-induced species invasions (Callaghan et al. 2004; Ims and Fuglei 2005; Post et al. 2009). In particular, the impact of climate on arctic vegetation may take place most abruptly when conveyed by changed dynamics of herbivores (Ims and Fuglei 2005; Post and Pedersen 2008) or pathogens (Olofsson et al. 2011).

## Electronic supplementary material

Below is the link to the electronic supplementary material.

**Online Resource 1.** Complementary description of the four plant communities defined by the hierarchical clustering. **Online Resource 2.** Photos illustrating a typical* Empetrum nigrum* die-back in the study region.(PDF 214 kb)
