# Supplementary material for: Outbreaks by canopy-feeding geometrid moth cause state-dependent shifts in understorey plant communities
Source: Oecologia. 2013 Apr 9;173(3):859–70. doi: 10.1007/s00442-013-2648-1 (PMC3824357; doi:10.1007/s00442-013-2648-1)

# ELECTRONIC SUPPLEMENTARY MATERIAL

## Outbreaks by canopy feeding geometrid moth cause state-dependent shifts in understorey plant communities

Karlsen, S.R.*, Jepsen, J.U., Odland, A., Ims, R.A., Elvebakk, A.

*Corresponding author: Stein Rune Karlsen, Norut, Northern Research Institute Tromsø, P.O.Box 6434 Tromsø Science Park, N-9294 Tromsø, Norway, tel.: +47 93419904, fax: +47 77629401, e-mail: stein-rune.karlsen@norut.no

****

**Online Resource 1.** Complementary description of the three plant communities defined by the hierarchical clustering. Indicator values in brackets.

***Crowberry-bilberry-dwarf cornel birch forest***

A birch forest characterized by multi-stemmed birch trees on dry soil and a field layer dominated by *C. suecicum* (0.296), *E. nigrum* (0.800) and *V. myrtillus* (0.323) to various degrees. The ground layer is rich in bryophytes (0.556) with species such as *Barbilophozia lycopodioides* (Wallr.) Loeske, *Dicranum* spp., *H. splendens*, and *P. schreberi*. The most closely related vegetation types in previous descriptions are the CoEM type in Hämet-Ahti (1963) and the fBpCsEhVm type in Karlsen et al. (2005). By dominance of *C. suecicium* the type is well separated from the widely distributed continental *E. nigrum* -lichen (*Cladina* spp.) birch forest type, which is not included in this study.

***Bilberry-dwarf cornel birch forest***

A birch forest type occurring on slightly more mesic and eutrophic soils than the previous one, where *V. myrtillus* (0.608) and C. *suecicum* (0.625) dominates the field layer and where *Avenella flexuosa* occurs in stable and low abundances.  *Empetrum nigrum* (0.133) is less pronounced, and the bryophyte (0.174) layer is less developed. The type is very common on the Varanger peninsula, and the most closely related vegetation types in previous descriptions are the CoM type in Hämet-Ahti (1963), and the fBpCsVm type in Karlsen et al. (2005).

### ***Low-herb birch forest***

The type is characterized by dominance of single-stem birches and a well-developed field layer. The field layer has a high species richness of low herbs, with *Geranium sylvaticum* (0.998) as the most abundant species. The low herbs *Ranunculus acris* (1.000), *Solidago virgaurea* (0.928) and *Viola biflora* (0.964) are common and abundant. Common bryophytes include *Barbilophozia* spp. and *Rhodobryum roseum* (Hedw.) Limpr., and *Climacium dendroides* (Hedw.) Web. & Mohr is locally dominant. The most closely related vegetation types in previous descriptions are the fBpGsRs type in Karlsen et al. (2005), and the type is a *Geranium* dominated variant of the widely distributed Meadow-heath forest described in Hämet-Ahti (1963).

## References


Hämet-Ahti L (1963) Zonation of the mountain birch forests in northernmost Fennoscandia. Annales Botanici Societatis Zoologicae Botanicae Fennica 34:1-127.

Karlsen SR, Elvebakk A, Johansen B (2005) A vegetation-based method to map climatic variation in the arctic-boreal transition area of Finnmark, north-easternmost Norway. Journal of Biogeography 32:1161-1186.

**Online Resource 2.** A typical *Empetrum nigrum* die-back in the study region. a) *E. nigrum* dominated plot showing withered plants with intact foliage one year after the moth outbreak, b) a similar plot several years after the outbreak, c) a close-up of withered *E. nigrum* foliage during a peak outbreak year. White arrows indicate autumnal moth larvae.

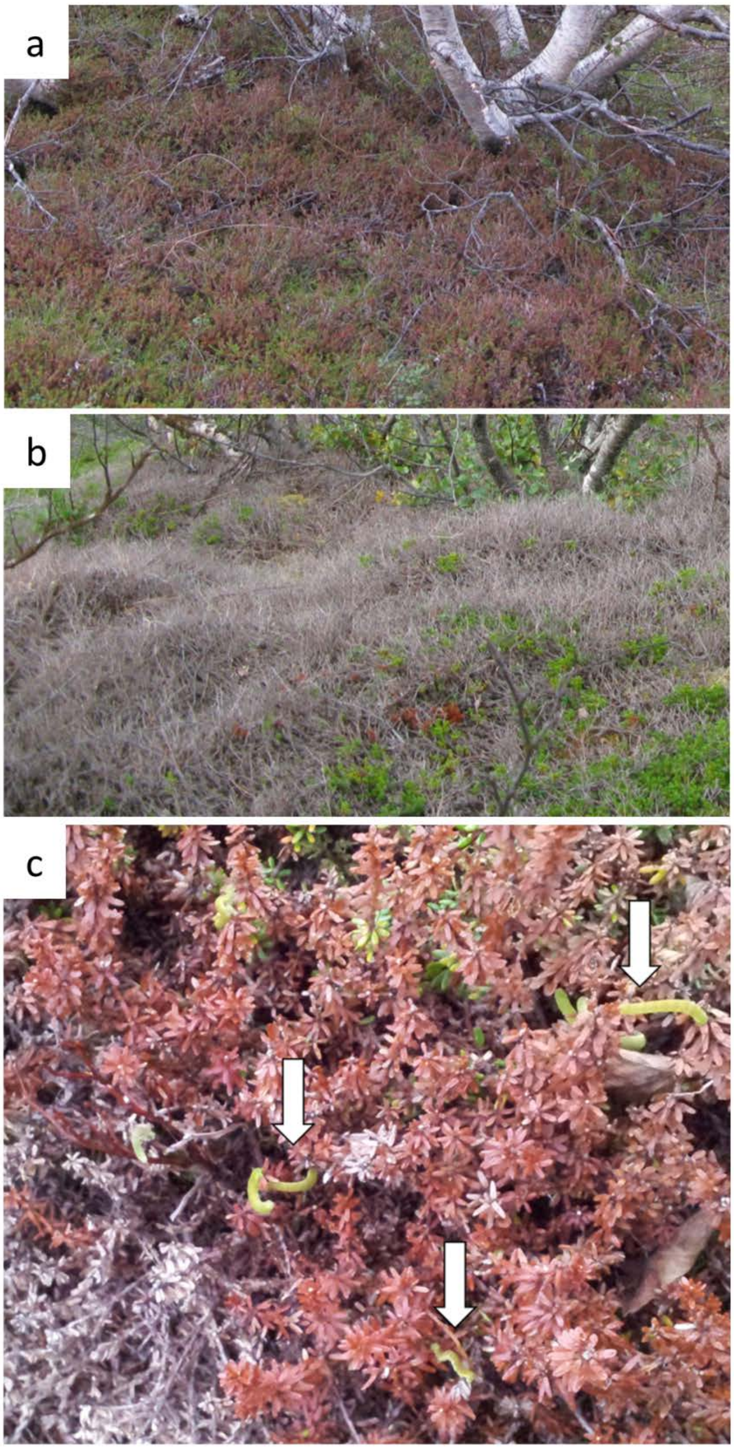

Supplement: Supplementary file 1 — Online Resource 1. Complementary description of the four plant communities defined by the hierarchical clustering. Online Resource 2. Photos illustrating a typical Empetrum nigrum die-back in the study region.(PDF 214 kb) [file 442_2013_2648_MOESM1_ESM.pdf]
